# Supplementary material for: Closing the Gap between the Auditory Nerve and Cochlear Implant Electrodes: Which Neurotrophin Cocktail Performs Best for Axonal Outgrowth and Is Electrical Stimulation Beneficial?
Source: Int J Mol Sci. 2023 Jan 19;24(3):2013. doi: 10.3390/ijms24032013 (PMC9916558; doi:10.3390/ijms24032013)
Supplement: Supplementary file 1 [file ijms-24-02013-s001.zip › ijms-2098138-suppl-FigureS1.pdf]

International Journal of *Molecular Sciences*

MDPI

Supplementary Materials

Closing the Gap between the Auditory Nerve and Cochlear Implant Electrodes. Which Neurotrophin Cocktail Performs Best for Axonal Outgrowth and is Electrical Stimulation Beneficial?

Dominik Schmidbauer[1], Stefan Fink[2], Francis Rousset[3], Hubert Löwenheim[2,4], Pascal Senn[3,5] and Rudolf Glueckert[1,6,*]

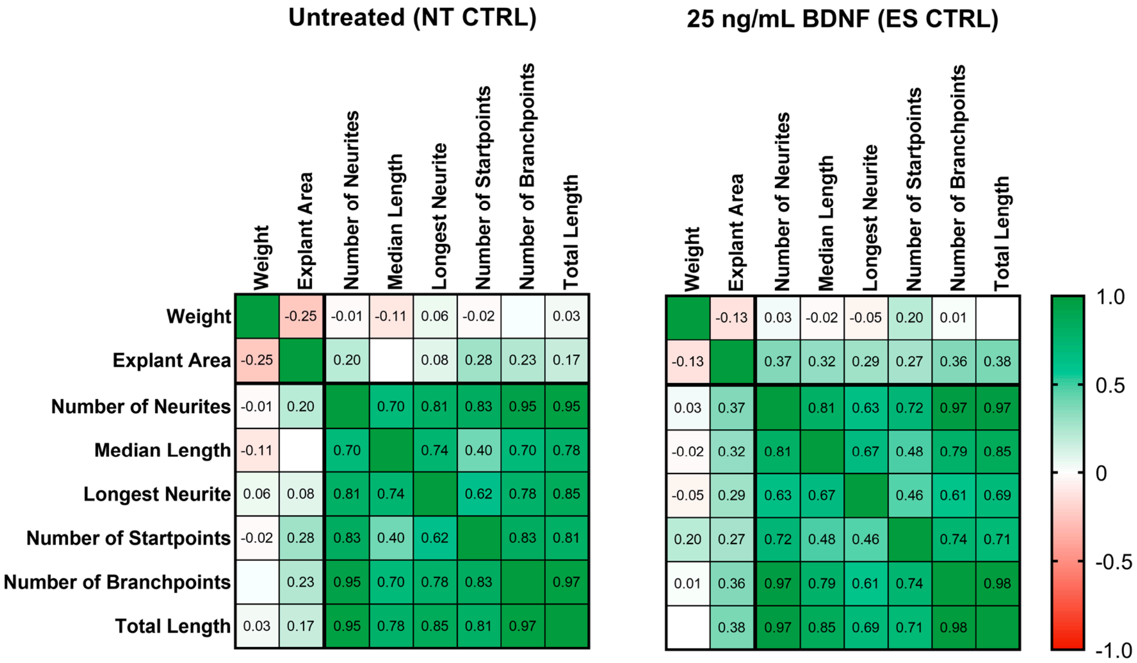

Supplementary Figure S1. Correlation matrices of discrete and continuous variables of untreated and BDNF-treated explants. The left matrix shows the correlations within the control group of the NT experiment, whereas the right matrix contains the correlations within the unstimulated, but with 25 ng/mL supplemented control group of the ES experiment. All correlation coefficients were computed by nonparametric Spearman's rank correlation analysis.
